# Cost to households in treating maternal complications in northern Ghana: a cross sectional study

**DOI:** 10.1186/s12913-014-0659-1

**Published:** 2015-01-22

**Authors:** Maxwell Ayindenaba Dalaba, Patricia Akweongo, Raymond Akawire Aborigo, Happiness Pius Saronga, John Williams, Gifty Apiung Aninanya, Rainer Sauerborn, Svetla Loukanova

**Affiliations:** University of Heidelberg, Institute of Public Health, Heidelberg, Germany; Navrongo Health Research Centre, Navrongo, Ghana; University of Ghana, School of Public Health, Accra, Ghana; Global Public Health, Monash University, Monash, Malaysia; Behavioural Sciences Department, School of Public Health and Social Sciences, Muhimbili University of Health and Allied Sciences, Dar es Salaam, Tanzania

**Keywords:** Maternal complication, Pregnancy, Economic burden, Household cost, Kassena-Nankana district, Ghana

## Abstract

**Background:**

The cost of treating maternal complications has serious economic consequences to households and can hinder the utilization of maternal health care services at the health facilities. This study estimated the cost of maternal complications to women and their households in the Kassena-Nankana district of northern Ghana.

**Methods:**

We carried out a cross-sectional study between February and April 2014 in the Kassena-Nankana district. Out of a total of 296 women who were referred to the hospital for maternal complications from the health centre level, sixty of them were involved in the study. Socio-demographic data of respondents as well as direct and indirect costs involved in the management of the complications at the hospital were collected from the patient’s perspective. Analysis was performed using STATA 11.

**Results:**

Out of the 60 respondents, 60% (36) of them suffered complications due to prolonged labour, 17% (10) due to severe abdominal pain, 10% (6) due to anaemia/malaria and 7% (4) due to pre-eclampsia. Most of the women who had complications were primiparous and were between 21–25 years old. Transportation cost accounted for the largest cost, representing 32% of total cost of treatment. The median direct medical cost was US$8.68 per treatment, representing 44% of the total cost of treatment. Indirect costs accounted for the largest proportion of total cost (79%). Overall, the median expenditure by households on both direct and indirect costs per complication was US$32.03. Disaggregating costs by type of complication, costs ranged from a median of US$58.33 for pre-eclampsia to US$6.84 for haemorrrhage. The median number of days spent in the hospital was 2 days - five days for pre-eclampsia. About 33% (6) of households spent more than 5% of annual household expenditure and therefore faced catastrophic payments.

**Conclusion:**

Although maternal health services are free in Ghana, women still incur substantial costs when complications occur and face the risk of incurring catastrophic health expenditure.

## Background

Despite recent evidence of reduction in maternal mortality worldwide, most developing countries still lack behind in achieving MDG 5, which seeks to reduce the maternal mortality ratio by 75% (i.e. 5.5% annual decline) between 1990 and 2015 [1-3]. Globally, every day, about 800 women die from pregnancy-related complications and almost all these deaths occur in developing countries [3]. Women living in developing countries are 300 times more likely to die as a result of childbirth or pregnancy-related complications than women living in developed countries [4]. About 80% of these deaths are due to severe bleeding (hemorrhage), infections, high blood pressure (pre-eclampsia and eclampsia) and unsafe abortion [3].

Women who survive life-threatening maternal complications are known as near misses. Near misses are more common than maternal deaths [5,6]. Globally, about 15% of pregnant women suffer from maternal complications [7,8]. Accumulated evidence shows that most of these complications occur during childbirth and the postpartum period [9]. Global efforts towards improving maternal health during this period have focused on increasing access to skilled care during child birth and providing emergency obstetric care where complications occur [3]. However, most deliveries in the developing world occur at home, which poses challenges when complications occur [10]. Utilization of health facilities for maternal services in these settings is hindered by several factors including cost. Anecdotal evidence suggests that even where maternal services are free, there may be unofficial or under-the-table payments and buying drugs outside the health facility when health facilities are out of stock. Also, indirect costs such as transportation, food and lodging can hinder the utilisation of maternal health care services at health facilities [11].

Although Ghana has instituted free maternal health care, Safe Motherhood programmes among others, an estimated 41% of deliveries are not attended by skilled health care providers [12]. Maternal health services covered under the free maternal health care initiative include antenatal care, delivery, caesarean section, obstetric complications and postnatal care [13,14].

Despite these interventions, the maternal mortality ratio is substantially high at 350/100,000 live births [15]. The incidence of maternal near misses has also been estimated within health facilities in urban Ghana to be 28.6 cases per 1,000 live births [12]. Cost of treating these complications have serious social and economic consequences for families [16-18]. However, beyond estimating the health burden, very few studies have estimated costs to patients or households in managing these complications [19,20]. Our study estimated the costs to households in the management of maternal complications based on referrals from peripheral health facilities to the next level of care (hospital).

## Methods

### Study site

The study was conducted in the Kassena-Nankana Districts (East and West) located in northern Ghana. For the purposes of this study, the two districts shall be referred to by their former name - the Kassena-Nankana District (KND). The KND has an area of about 1,675 square kilometres with a population of about 152,000 people [21]. Subsistence agriculture is the mainstay of the people. The district is characterized by a high poverty and mortality burden. The district is in one of the poorest regions in Ghana with poverty incidence of 88% [22,23]. Maternal mortality ratio for the period 1995–1996 was estimated at 637/100,000 live births but it declined to 373 maternal deaths per 100,000 live births based on an estimate for the period 2002–2004, representing a 40% reduction in the ratio [24].

With regards to health care, the KND has a district hospital located in the capital town (Navrongo) that serves as a referral point for all health facilities in the district. The hospital is the only health facility equipped to offer comprehensive emergency obstetric care in the district [11]. There are six health centres, one private clinic and twenty seven Community-based Health Planning and Services (CHPS) compounds. The CHPS initiative started in 1999 by the government of Ghana with the aim of increasing access to primary health care in the entire country. In this initiative, midwives and community health nurses are trained and sent to rural communities to provide basic preventive and curative services as well as doorstep services. These include antenatal care, delivery and postnatal services [25].

### Study design and data collection

A cross sectional quantitative survey design was employed in data collection. Data was collected between February and April 2014 from the patient’s perspective. Two graduate research officers conducted all the interviews after two weeks of training on the study tools. Women with pregnancy-related complications were defined as women who were diagnosed by health staff during pregnancy or delivery to have a maternal complication and were referred from the health centre to the hospital for treatment. Data on all women who had pregnancy-related complications at the six main health centres in the district between April 2012 and March 2013 (12 month period) were obtained from the six health centres.

A total of 296 women with maternal complications were referred from the health centres to the hospital within the period. However, contact information for 145 cases were never recorded by the health centre and therefore could not be traced in the community. In addition, 91 women had migrated from the district when their homes were visited. Thus only 60 women who were met during our visits were interviewed. Information on socio-demographic characteristics, direct and indirect costs of treating the complications were obtained. The reasons for referral reported by the women were also obtained. Since the aim was to capture official and unofficial payments made by women, all expenditures incurred within the hospital and outside the hospital were included.

This study was part of a larger project (QUALMAT project) which aimed to improve quality of maternal and prenatal care in Ghana, Tanzania and Burkina Faso by testing two interventions: a computer-assisted clinical decision support system and performance-based incentives for improvement of the quality of maternal health services provided [26,27].

### Ethics statement

Ethical approval was obtained from the ethics committee of the University of Heidelberg (S-173/2008) and the Institutional Review Board of the Navrongo Health Research Centre in Ghana (NHRCIRB 085) before the study was conducted. In addition, individual oral informed consent was obtained from respondents before being interviewed.

### Data processing and analysis

Data were entered into Epidata 3.1 and exported into STATA 11.0^©^ for analysis. Descriptive analysis on background characteristics of respondents was done. The direct out-of-pocket costs for each pregnancy-related complication were estimated. Direct and non-direct medical costs were estimated by summing the costs and means calculated. The direct medical costs covered out-of-pocket payments for drugs, laboratory tests and medical supplies. Direct non-medical costs included all expenditure made on food during the health seeking process and transportation to the hospital and back home. The transportation cost included both the woman and the person who accompanied her to the hospital. Indirect costs associated with productivity lost were estimated by multiplying the number of days spent at the hospital by the daily minimum wage for the year 2013 (GH¢5.24/US$2.8). This was calculated for both the patient and the caretaker. Pre-referral costs were not collected and for that matter are not part of the analysis. Given that health centres are generally closer to the people, indirect cost such as transportation will be negligible. Also health centres do not have in-patient services hence costs related to in-patient care will be marginal.

To determine the financial impact of maternal complications for households, actual cost incurred by the household was measured in relation to average annual household expenditure obtained from the Ghana Living Standards Survey Report of the fifth round (GLSS 5)_ Gh¢1,918 (US$1009) [28]. Catastrophic Health Expenditure (CHE) was also assessed. CHE is when a household’s out-of-pocket (OOP) payments are so high relative to its available resources which would require the household to forego the consumption of other essential goods and services [29]. Thus total OOP health care payments in excess of a certain threshold of household resources (household income, expenditure or consumption) are catastrophic. There is no consensus regarding the threshold for defining catastrophic health expenditures. Most authors have used threshold levels of 2.5%, 5%, 10% 15% and 20% of total household income.

All costs were collected in Ghana Cedis (GH¢) and results presented in US$. The US$ conversion was based on the average exchange rate for 2013 (1US$ = 1.9GH¢). Given that the numbers interviewed were small, results on expenditures are presented in median.

## Results

### Socio-demographic characteristic of respondents

Of the 60 women, majority were married 88% (53). The mean age was 26 years (median = 25; range = 16–39 years) with standard deviation of 6. Most of the women 34% (20) were between 21–25 years, 23% (14) were above 30 years and 23% (14) were less than 20 years old. Majority 57% (34) of the respondents were farmers/traders, 25% (15) were unemployed, 10% (6) were employed in the formal sector, 5% (3) were artisans and 3% (2) were students. In terms of education, 56% (34) of the respondents had basic level education (primary and junior secondary level), 22% (14) had post-basic education (secondary education or higher), and 22% (13) had no formal education. Majority of the women who had complications were primiparous 47% (28) (Table 1).

**Table 1:** **Socio-demographic characteristic of respondents**

**Category**	**Sub-category**	**Frequency**	**Percentage (%)**
Age	20 and below years	14	23
	21-25 years	20	34
	26-30 years	12	20
	Above 30 years	14	23
Marital status	Married	53	87
	Not married	8	13
Ethnicity	Kassena	28	47
	Nankana	32	53
Occupation	Unemployed	15	25
	Trader/farmer	34	57
	Artisan	3	5
	Formal sector employee	6	10
	Student	2	3
Education	No formal education	13	22
	Primary school	20	33
	Junior high school	14	23
	Senior high school	8	14
	Tertiary school	5	8
Parity	1	28	47
	2	16	27
	3	13	22
	4	2	3
	5	1	2

### Type of complication and source of treatment

As shown in Table 2, more than half (60%) of the pregnancy-related complications reported by respondents resulted from prolonged labour (labour lasting more than 12 hours). About 17% (10) was due to severe abdominal pain, 10% (6) due to anaemia/malaria, and about 7% (4) due to pre-eclempsia. About 80% (49) of respondents sought care at a hospital and the remainder at a regional hospital.

**Table 2:** **Distribution of pregnancy complications**

**Type of complication**	**Frequency**	**Percent (%)**
Pre-eclampsia	4	6.7
Anaemia/malaria	6	10
Haemorrhage	2	3.3
Infection	2	3.3
Abdominal pains	10	16.7
Prolong labour	36	60
Total	60	100

### Cost of treating pregnancy complications

**Figure 1:**
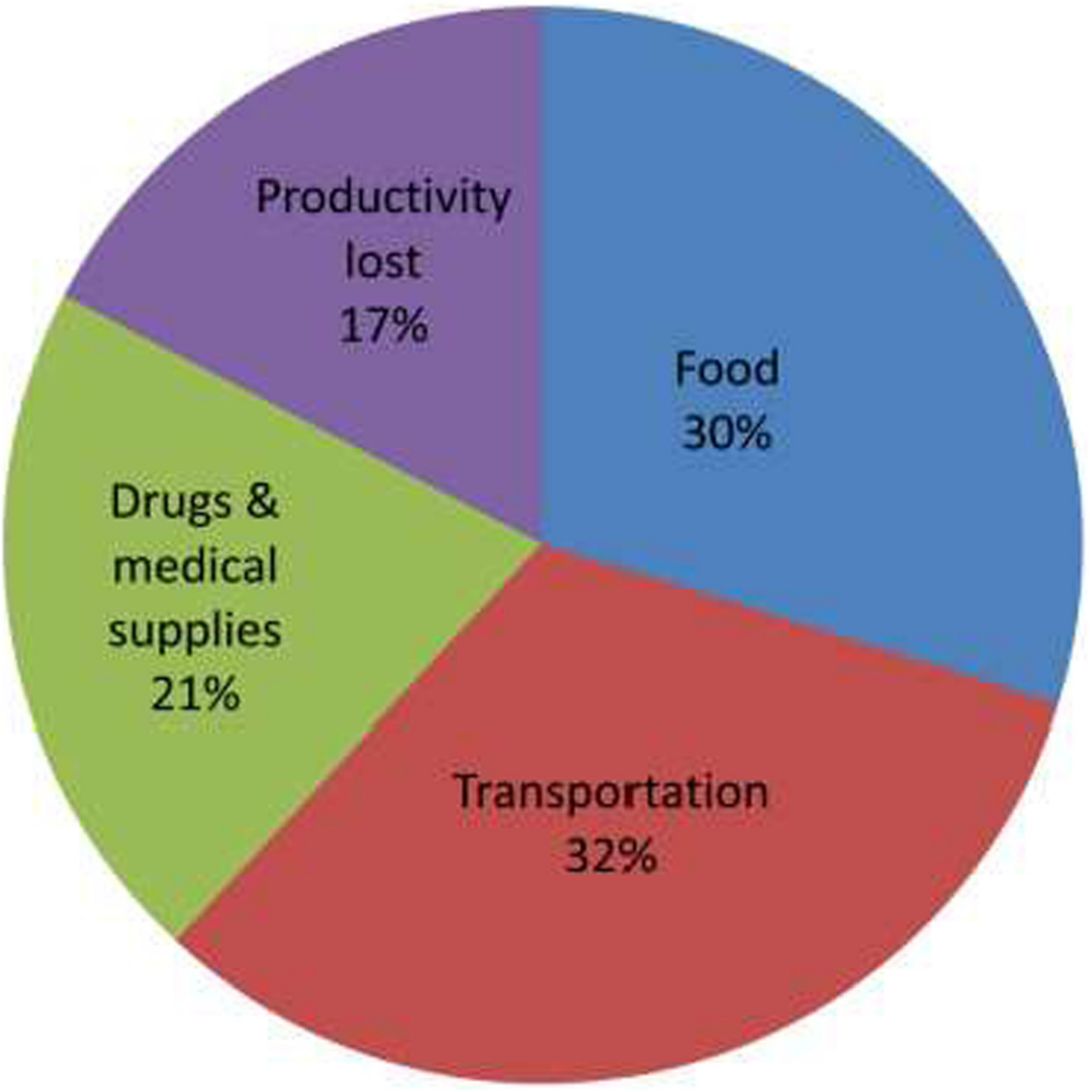
**Cost components as a proportion of total cost.**

Table 3 presents the main cost components for each of the five maternal complications. Of the 60 women who had complications, only 30% (18) incurred direct medical costs and 70% (42) reported zero direct medical cost. Out of the 18 women, the total direct medical cost emanating from drugs, laboratory services and scanning was US$543.68 and the median cost was US$8.68(IQR = 43.68) per treatment. Thus, 21% of the total cost of treatment was spent on direct medical costs (Figure 1). Most of these direct medical costs were incurred outside the hospital because of shortage/non-availability of prescribed drugs or non-availability of equipment. For instance, 2 years preceding the survey, the Navrongo hospital operated without an ultrasound scan. Accordingly, women had to obtain their scans from private sources which made them incur additional costs.

**Table 3:** **Cost of treating pregnancy complications by type of complication (US$)**

**Type of complication**	**Variables**	**Food (US$)**	**Transportation (US$)**	**Drugs & medical supplies (US$)**	**Productivity lost (US$)**	**Total cost (US$)**
Pre-eclampsia	Observation	4	4	3	4	4
	mean	24.87	10.13	39.82	14.48	79.35
	median	26.05	10.00	7.89	15.17	58.33
	IQR	16.58	13.42	98.95	9.65	64.65
	Total	99.47	40.53	119.47	57.92	317.39
Aneamia/Malaria	Observation	6	6	4	6	6
	mean	22.89	13.12	27.11	13.33	67.42
	median	14.21	14.63	28.95	8.27	56.76
	IQR	9.47	9.47	35.26	5.52	39.87
	Total	137.37	78.74	108.42	79.98	404.51
Haemorrhage	Observation	0	2	0	0	2
	mean	0	6.84	0	0	6.84
	median	0	6.84	0	0	6.84
	IQR	0	6.32	0	0	6.32
	Total	0	13.68	0	0	13.68
Infection	Observation	1	2	0	1	2
	mean	18.95	15.89	0	11.03	30.88
	median	18.95	15.89	0	11.03	30.88
	IQR	0.00	0.21	0	0	30.19
	Total	18.95	31.79	0	11.03	61.77
Abdominal pains	Observation	8	10	4	8	10
	mean	11.25	19.37	20.13	6.55	41.66
	median	11.84	20.53	7.37	6.89	38.67
	IQR	11.84	27.37	32.37	6.89	50.25
	Total	90.00	193.68	80.53	52.40	416.61
Prolong labour	Observation	32	36	7	32	36
	mean	13.62	13.40	33.61	7.93	39.09
	median	9.47	12.89	6.32	5.52	28.41
	IQR	11.84	15.26	60.53	6.89	37.09
	Total	435.79	482.32	235.26	253.73	1407.09
Total	Observation	51	60	18	51	60
	mean	15.33	14.01	30.20	8.92	43.68
	median	9.47	13.58	8.68	5.52	32.03
	IQR	14.21	16.05	43.68	8.27	42.55
	Total	781.58	840.74	543.68	455.05	2621.05

The median transportation cost was US$13.48 (IQR = 16.05) per person (patient and person accompanying the patient) representing 32% of the total cost (Figure 1). Majority of the respondents were transported to the hospital with the official vehicle of the health centre (37%). Respondents reported paying between US$11 and US$13 to fuel the vehicle to the referral point. The median expenditure made on food for both patient and caretaker was estimated at US$9.47(IQR = 14.21) per person. In addition, median indirect cost attributed to productivity losses was estimated at US$5.2 (IQR = 8.27) per person.

Overall, the median expenditure made by households on both direct and indirect costs per complication was US$ 32.03(IQR = 42.55) per person. Indirect costs accounted for the largest proportion of total cost (79%). Disaggregating costs by type of complication, women who had pre-eclampsia spent more (median = US$58.33; IQR = US$64.65) than those who had other types of complications. Women with haemorrhage incurred the least cost (median = US$6.84; IQR = 6.32).

As shown in Table 4, the median number of days spent in the hospital was two days (mean = 3 days, IQR = 3). The median days varied from zero for haemorrhage to six days for pre-eclampsia.

**Table 4:** **Mean number of days admitted at the hospital**

**Type of complication**	**Frequency**	**Mean (days)**	**Median (days)**	**Interquartile range (IQR)**
Pre-eclampsia	4	5	6	3.5
Aneamia/malaria	6	5	3	2
Haemorrhage	2	0	0	0
Infection	2	2	2	4
Abdominal pains	10	2	2	2
Prolong labour	36	3	2	2
Total	60	3	2	3

### Catastrophic health expenditure

Table 5 presents costs of maternal complication as a proportion of annual household expenditure for 2008 (US$1009). Payments for maternal complications amounted to about 3% of annual household expenditure. Pre-eclampsia and anemia/malaria accounted for the greatest burden on households (6% of annual household expenditure). Haemorrhage accounted for the least burden on households (1% of annual household expenditure). About 33% (6) of households spent more than 5% of annual household expenditure and therefore faced catastrophic payments (threshold at 5%).

**Table 5:** **Median cost as a percentage of annual household expenditure**

**Type of complication**	**Median cost (US$)**	**Percentage of annual household expenditure (%)**
Pre-eclampsia	58.33	6
Aneamia/Malaria	56.76	6
Haemorrhage	6.84	1
Infection	30.88	3
Abdominal pains	38.67	4
Prolong labour	28.41	3
Total	32.03	3

### Limitation of the study

One limitation was the possibility of recall bias. Some expenditure could either have been overestimated or underestimated. We however recognize that health expenditure and hospitalization are critical events for low income families and therefore can easily be recollected. Field staff training also put emphasis on probing strategies to help mitigate the effect of recall bias.

Also, the study could not contact about 49% (145) of all cases due to the absence of contact details. It is worth noting that, as in many developing countries, home addresses in Ghana is a major challenge. However, the health centres are located within a demographic surveillance system which has developed a unique home address system with identity cards for all individuals in the district. This should have facilitated the recording of contact information which is part of the standard of care within the health care system. The nurses at the health centres however complained that most patients do not come with their identity cards and recalls often led to wrong addresses.

We did not include intangible costs in our analysis which therefore underestimated our cost results. Intangible costs relates to the reduced quality of life due to illness. It includes pain, psychological pressure, reduced joy of life and social prestige due to the illness [30]. However, this cost component, though important, is difficult to measure and is therefore not usually included in cost studies.

## Discussion

The results of this cross-sectional study showed that the economic burden of pregnancy complications to households (median = US$32.03) was high. This constitutes a large expenditure for households. Given a minimum monthly wage of about US$60 in Ghana, it implies that the amount spent on complications represented more than halve the monthly minimum wage earned by Ghanaians. In other words, expenditure for maternal complications amounted to about 3% of annual household expenditure. In fact, about 33% (at 5% threshold level) of the study participants faced catastrophic health expenditures. Given that the study district is in one of the poorest regions in Ghana with poverty incidence of 88% [22,23] suggests that majority of participants were already vulnerable to even the smallest expenditure on health care .As it has been reported elsewhere, household expenditure on pregnancy-related complications will not only deplete household resources, especially poor households, but also the household’s ability to meet subsistence needs and therefore lead to poverty or deeper poverty [16-18]. Cost of seeking maternal care may prevent or delay women in seeking care [31], leading to deteriorating health, increased expenses and possible death. Although our study could not establish the magnitude of long term effects of complications, previous studies have revealed that women who experience maternal complications can suffer from other long term severe consequences and increase their risk of death [4,18,32].

The cost obtained in this study is lower than the cost of US$92 estimated in a similar study conducted in southern Ghana between 1999 and 2000 in hospitals [20]. The main reason for this difference could be due to the free maternal health care initiative introduced in Ghana in 2004 [33]. With the free maternal health policy, all direct medical costs incurred in all public and accredited private health facilities are borne by the government.

Indirect costs constituted a major cost component (79%). This is worrying given that indirect costs are not covered by the free maternal health policy. Indirect costs such as transportation cost has been identified in many studies as a major obstacle to utilization of maternal health services [14,34-36]. In most poor rural settings such as the KND, free ambulance services are non-existent and social support schemes for the vulnerable are rare and yet provide the only viable option in the face of persistent government failures to respond to the needs of the population.

Our findings raise serious concerns about the context in which the free maternal health policy is being implemented. Maternal health services are not free if cost of medicines and services are still directly borne by patients. The policy can be deemed to have failed to ensure that cost of health services is not a barrier to access to emergency obstetric services. Women in the KND and others in similar settings still suffer huge financial risks in accessing maternal health services and the health system needs to respond appropriately. Our results are supported by Borghi et al. [20] who found similar expenditures in the southern part of the country.

The findings expose a critical limitation in the health system especially in preparing for maternal emergencies in referral facilities. A functional health system should maintain adequate and regular supply of essential medical supplies and equipment at referral hospitals. Current conditions at referral facilities however suggest otherwise, thus endangering the lives of women. Interventions such as the provision of essential maternal health care services, free maternal health care, maternity referral system have the propensity to reduce significantly, costs and consequences of pregnancy-related complications. However, implementation has been a major challenge [33,37], thus limiting their potential impact. This may be one of the critical challenges within health systems in the developing world that have slowed down progress towards achieving MDG5. More pragmatic efforts are urgently needed to improve implementation of these interventions including non-medical interventions such as ambulance services so as to reduce the financial burdens to women seeking emergency obstetric care.

## Conclusions

Although, officially, maternal health services are free in Ghana, women in need of emergency obstetric care in formal health care facilities incur substantial costs and face the risk of incurring catastrophic health expenditure. The current health system within which the free maternal health service policy is being implemented is necessary but not sufficient to reduce the economic burden of treating maternal complications and to provide adequate financial protection to households. Enacting a policy without necessarily providing the enabling environment to maximize the outcomes puts a dent on the health system. In view of this we recommend the following:

1. That health facilities should be adequately equipped with both diagnostics and essential medicines to make the free maternal health care policy a reality.
2. That the provision of ambulance services and the inclusion of transportation costs in the free maternal health service policy may yield some dividends.
3. That a system for replenishing expenses related to ambulance services be instituted within the maternal health care policy to make it sustainable.
4. That poverty alleviation programmes that focus on women have the potential to make poor households resistant to health financial shocks and thus reduce the economic burden of treating illness and the risk of incurring catastrophic health expenditures due to maternal complications.
5. That more public education is required to encourage patients to carry their personal identity cards or house addresses when visiting health facilities in order to facilitate follow up visits.
